# Dexamethasone Promotes Autophagy Dependent Ferroptosis of Placental Trophoblast Cells Through GRα

**DOI:** 10.1111/jcmm.70613

**Published:** 2025-07-07

**Authors:** Junlei Lu, Xinyun Huang, Yuan Xu, Qiaoping Xu, Hongkai Shang

**Affiliations:** ^1^ The Fourth Clinical School of Medicine Zhejiang Chinese Medical University Hangzhou China; ^2^ Zhejiang University Hangzhou China; ^3^ Xiaoshan District Second People's Hospital Hangzhou China; ^4^ Affiliated Hangzhou First People's Hospital, School of Medicine Westlake University Hangzhou China

**Keywords:** autophagy, dexamethasone, ferroptosis, placental trophoblast cells, premature infant

## Abstract

Dexamethasone (DEX) has been extensively employed for the treatment of pregnancy disorders related to preterm birth. However, the precise mechanism by which prenatal DEX exposure affects foetal development remains unclear. This study delves into investigating the influence of DEX and ferroptosis inhibitors on the activity and ferroptosis level of placental trophoblast cells through various in vitro tests. The results demonstrated that prenatal administration of DEX led to a substantially increased ferroptosis level in placental tissues. DEX decreased cell viability, increased iron accumulation and increased lipid peroxidation. Treatment with ferroptosis inhibitors reversed these effects. Mechanistically, DEX regulated autophagy and modulated the protein levels of AMPKα/BECN1 and ATG5/ATG7/NCOA4 signalling pathways by inhibiting its receptor GRα. Additionally, autophagy activation mediated through the AMPKα/BECN1 axis and ferritin degradation via ATG5/ATG7/NCOA4 signalling was associated with DEX‐induced ferroptosis. Overall, this study provides experimental evidence supporting the detrimental side effects of DEX on preterm pregnancy‐related disorders. It identified that AMPKα/BECN1 and ATG5/ATG7/NCOA4 were key pathways by which DEX induced autophagy‐dependent ferroptosis in placental trophoblast cells.

## 1 Introduction

Dexamethasone (DEX) is a synthetic glucocorticoid known for its ability to promote lung maturation and reduce premature infant mortality [1]. As the risk of preterm birth is nearly 10% in pregnant women, a large proportion of pregnant women receive glucocorticoid therapy [2]. The rate of synthetic glucocorticoid treatment is 54% according to a World Health Organisation survey of maternal and infant health including 359 institutions from 29 countries and 91% in several countries [3]. However, the utilisation of prenatal DEX remains a subject of controversy. An increasing number of studies have confirmed that prenatal DEX administration reduces foetal birth weight and triggers developmental toxicity, leading to diseases in various organs in adult offspring [4, 5]. Simultaneously, glucocorticoid can interfere with the function of trophoblast cells in early pregnancy. In rats, glucocorticoid exposure from gestational days 7–13 induced preeclamptic symptoms, in part due to the inhibition of trophoblast invasion [6]. Previous studies have demonstrated that DEX, a commonly prescribed medication during pregnancy, exhibits toxicity to foetal development, potentially due to its effects on the proliferation of placental trophoblast cells [5, 7]. Despite its common use, the precise action of DEX on trophoblast cells and its underlying molecular mechanisms remain not fully understood.

As reported, DEX can induce ferroptosis of osteoblasts [8, 9]. Ferroptosis, an emerging necrotic form of programmed cell death (PCD), is characterised by phospholipid peroxidation in plasma membranes caused by reactive oxygen species (ROS) produced during free iron‐mediated Fenton reactions [10]. Ferroptosis in placental trophoblast cells may lead to placental dysfunction, which in turn can contribute to the occurrence and progression of placenta‐related diseases such as pre‐eclampsia, foetal growth restriction, preterm birth and abortion [11, 12, 13]. Our previous research has demonstrated that DEX can activate ROS production, leading to a loss of mitochondrial membrane potential, ultimately leading to apoptosis in human placental JEG‐3 cells and inhibit placental growth [14]. However, the specific mechanism by which DEX affects ferroptosis of trophoblast cells needs further clarification

Although ferroptosis was considered independent of autophagy in the early days, recent evidence indicates that autophagy is closely related to ferroptosis [15]. Autophagy has been found to be an upstream mechanism of ferroptosis through the regulation of intracellular iron balance and lipid peroxidation. Moreover, the depletion of core components of autophagic machinery, such as autophagy‐related 5 (ATG5), autophagy‐related 7 (ATG7), and beclin 1 (BECN1) has been found to inhibit cell ferroptosis [16]. Recently, nuclear receptor coactivator 4 (NCOA4)‐mediated ferritinophagy is increasingly recognised as a crucial mechanism in modulating ferroptosis. Upon immoderate activation, there is a direct outbreak of ferroptosis via superabundant iron producing the Fenton reaction [17]. Interestingly, ROS‐induced lipid peroxidation contributes to autophagy and ferroptosis since excessive ROS disrupts bio‐membranes and promotes lipid peroxidation chain reactions, leading to mitochondrial dysfunction and subsequent cell death [18]. Recent studies have unveiled increased autophagy in placentas from pregnancies complicated by preeclampsia or foetal growth restriction [19, 20]. Additionally, several studies have demonstrated that DEX contributes to the occurrence and development of various diseases by inducing cell autophagy, including placental growth [21], male infertility [22] and multiple cancers [23, 24]. However, the potential relationship between DEX‐induced autophagy and ferroptosis remains unclear.

While natural glucocorticoids (cortisol and corticosterone) bind to both the glucocorticoid receptor (GR) and mineralocorticoid receptor (MR) to orchestrate cellular outcomes, synthetic glucocorticoids such as DEX act exclusively by solely binding to the GR [25]. Glucocorticoid receptor α (GRα) is the predominant and functionally active receptor. GRα isoforms function as dose‐dependent transcription factors, overseeing various transcript sets directly or indirectly in a tissue‐specific manner [26]. Recent publications showed that the human placenta expresses multiple glucocorticoid receptor isoforms, including GRα [27]. Previous studies have demonstrated that the activation capability of DEX on the AMP‐activated protein kinase (AMPK) signaling pathway is contingent on GRα [28]. The AMPK signalling pathway is intricately linked to autophagy‐dependent ferroptosis. AMPKα‐mediated BECN1 phosphorylation triggers LC3B puncta accumulation and autophagy, potentially promoting ferroptosis [29]. ALOX5 promotes autophagy‐dependent ferroptosis by activating the AMPK/mTOR pathway in melanoma [30]. Furthermore, activation of the AMPK–mTOR pathway by SIRT3 promotes autophagy‐dependent ferroptosis of trophoblast cells [31]. Hence, our study aims to investigate the effect of DEX on autophagy‐dependent ferroptosis of placental trophoblast cells and elucidate its potential molecular mechanism, with a particular focus on AMPKα/BECN1 and ATG5/ATG7/NCOA4 signalling pathway.

## 2 Materials and Methods

### 2.1 Clinical Samples and Ethics

Participants were enrolled from Hangzhou First People's Hospital affiliated with Zhejiang University School of Medicine, between October 2021 to January 2023. The participants were divided into two groups: the control group (*n* = 5) and the DEX group (*n* = 5). The control group consisted of women who experienced natural pre‐term birth between 28 weeks + 0 days and 36 weeks + 6 days of pregnancy. In contrast, the DEX group consisted of women who received a course of DEX (6 mg intramuscular injection, repeated once every 12 h, for a total of four times) treatment within 7 days prior to preterm birth. Table 1 displays the clinical characteristics of the pregnancy. Placental tissue samples (5 × 5 × 5 mm^3^) were obtained within 15 min postpartum. Subsequently, the tissues were rinsed in cold PBS to remove maternal and foetal blood. The placental tissues were snap frozen in liquid nitrogen for 10 min and were then stored at −80°C until use. All participants signed informed consent forms. The study received approval from the Human Ethics Committee of Hangzhou First People's Hospital. The procedures used in this study comply with the principles of the Declaration of Helsinki.

**TABLE 1:** Sample source personal information.

	Control	DEX	*p* (chi‐squared test)
Age/year	29.6 ± 5.4	27.6 ± 2.4	0.197
Weight/kg	64.7 ± 6.9	72.9 ± 29.7	0.35
High/m	1.60 ± 0.01	1.62 ± 0.05	0.306
BMI	25.3 ± 2.9	27.8 ± 11.2	0.35
Gestational weeks	35.7 ± 1.7	35.5 ± 1.3	0.423
DEX treatment weeks	NA	35.0 ± 1.3	NA

### 2.2 Prussian Blue Staining

The Prussian Blue Staining Kit was purchased from ServiceBio (G1029, Wuhan, China). Placental tissues paraffin sections (thickness = 10 μm) were prepared and immersed successively in xylene I, xylene, anhydrous ethanol I, anhydrous ethanol and ethanol for 20, 20, 5, 5 and 5 min, respectively. The sections were then washed three times in distilled water. For Prussian blue staining, the proportions of Prussian blue dye solution A and Prussian blue dye solution B were mixed into the Prussian blue staining solution. The sections were stained in this solution for 1 h, followed by washing twice in distilled water. Prussian blue staining solution C was applied to the sections for 3 min before they were rinsed in running water. The sections were dehydrated and sealed as follows: sections were placed into anhydrous ethanol I (for 5 min), anhydrous ethanol II (for 5 min), anaerobic ethanol (for 5 min), xylene I (for 5 min), xylene (for 5 min) and finally a neutral gum sealing sheet. Under acidic conditions, the high iron salt in the section acted with potassium ferrocyanide to generate iron ferrocyanide. Prussian blue staining enables observation of the presence of iron in tissue cells and the location and content of iron elements. Iron and hemosiderin were stained blue and nuclei were stained red.

### 2.3 Immunohistochemistry

The expression of 4‐Hydroxynonenal (4‐HNE) in placental tissues was assessed by the immunohistochemical (IHC) method. After heating, dewaxing and rehydrating, the sections were immersed in sodium citrate buffer (pH = 6.0) for antigen retrieval. Subsequently, the sections were immersed in 3% hydrogen peroxide to inhibit endogenous peroxidase activity. After three washes, the sections were incubated with the primary antibody (4‐Hydroxynonenal, Thermo Fisher, MA5‐27570, 1: 100, USA) overnight at 4°C. The next day, sections were incubated with the secondary antibody (ServiceBio, GB23301, 1: 200, China) for 40 min at 37°C. Each section was immersed in 500 μL of diaminobenzidine working solution for 3–10 min at room temperature.

### 2.4 Cell Culture

HTR‐8/SVneo cells (Human Trophoblast cells) were purchased from Ningbo Mingzhou Biotechnology Co., LTD. TEV‐1 cells (Human Trophoblast cells) were purchased from Shanghai Yaji Biotechnology Co., LTD. HTR‐8/SVneo cells were maintained in RPMI‐1640 medium (procell, PM150110, Wuhan, China) supplemented with 10% foetal bovine serum (FBS) (procell, 1641010‐500, Wuhan, China), 100 μg/mL streptomycin and 100 U/mL penicillin (procell, PB180120, Wuhan, China) under standard culture conditions (37°C and 5% CO_2_ incubator). Similarly, TEV‐1 cells were maintained in DMEM medium (procell, PM150220, Wuhan, China) supplemented with 10% foetal bovine serum (FBS) (procell, 1641010‐500, Wuhan, China), 100 μg/mL streptomycin and 100 U/mL penicillin (procell, PB180120, Wuhan, China) under standard culture conditions (37°C and 5% CO_2_ incubator). HEK293T cells were procured from Wuhan Punocai Life Technology Co., LTD. (CL‐0005, Wuhan, China), and were cultured in DMEM medium (procell, PM150220, Wuhan, China) supplemented with 10% foetal bovine serum (FBS) (procell, 1641010‐500, Wuhan, China), 100 μg/mL streptomycin and 100 U/mL penicillin (procell, PB180120, Wuhan, China) under standard culture conditions (37°C and 5% CO_2_ incubator).

### 2.5 Cell Transfection

Cells in the logarithmic growth phase were seeded into a 24‐well plate (5 × 10^4^ per well) and incubated overnight at 37°C in a 5% CO_2_ incubator. Two hours prior to transfection, the medium was changed to a serum‐free culture medium. The transfection procedure is outlined as follows: 20 μL of siRNA (si‐NC, si‐AMPKα, si‐BECN1, si‐ATG5, si‐ATG7, si‐NCOA4, sangon, Shanghai, China) or 2 μg plasmid (vector, GRα OE, vectorbuilder, Guangzhou, China) was diluted in 100 μL of serum‐free opti‐MEM (Gibco, 31985062, Shanghai, China) before being mixed and incubated at room temperature for 5 min. Next, 3 μL of Lipofectamine 2000 (Invitrogen, 11668019, Shanghai, China) was added to 100 μL of opti‐MEM and incubated at room temperature for 5 min. Lipofectamine 2000 was combined with the siRNA or plasmid mixture, followed by incubation at room temperature for 15 min. Each well received 200 μL of the mixture. After mixing, the cells were cultured in a 5% CO_2_ incubator at 37°C. After 6 h, the mixture was aspirated and replaced with a normal culture medium for further culture.

### 2.6 Cell Treatments

The cell experiment was divided into five parts. In the initial segment, cells were randomly assigned into four groups: (A) Control group: conventional culture; (B) DEX group: cells were treated with DEX (1 μM; Sigma, D4902, Shanghai, China) for 24 h; (C) DEX+Ferrostatin‐1 group: cells were pre‐treated with ferroptosis inhibitor (Ferrostatin‐1; 10 μM; sigma, SML0583, Shanghai, China) for 1 h before DEX; (D) DEX+DFOM group: cells were pre‐treated with deferoxamine (DFOM; 100 μM; sigma, PHR3411, Shanghai, China) for 1 h before DEX. In the second part, cells were randomly categorised into four groups: (A) Control group; (B) DEX group; (C) DEX+vector group: cells were transfected with empty pcDNA3.1 plasmid (vector) for 48 h, followed by DEX treatment; (D) DEX+GRα OE group: cells were transfected with GRα‐overexpressed plasmid (GRα OE) for 48 h, followed by DEX treatment.

In the third part, cells were randomly divided into nine groups: (A) Control group; (B) DEX group; (C) DEX+CC group: cells were treated with AMPK inhibitor compound C (CC; 10 μM; MCE, HY‐13418A, Shanghai, China) for 24 h, followed by DEX treatment; (D) DEX+si‐NC group: cells were transfected with negative control si‐RNA for 48 h, followed by DEX treatment; (E) DEX+si‐AMPKα group: cells were transfected with si‐AMPKα for 48 h, followed by DEX treatment; (F) DEX+si‐BECN1 group: cells were transfected with si‐BECN1 for 48 h, followed by DEX treatment; (G) DEX+si‐ATG5 group: cells were transfected with si‐ATG5 for 48 h, followed by DEX treatment; (H) DEX+si‐ATG7 group: cells were transfected with si‐ATG7 for 48 h, followed by DEX treatment; (I) DEX+si‐NCOA4 group: cells were transfected with si‐NCOA4 for 48 h, followed by DEX treatment. In the fourth part, cells were randomly divided into eight groups: (A) Control group; (B) Erastin group: cells were treated with ferroptosis inducer erastin (20 μM; sigma, 329,600, Shanghai, China) for 24 h; (C) Erastin+CC group: cells were treated with CC (10 μM) for 24 h, followed by erastin treatment; (D) Erastin+si‐NC group: cells were transfected with si‐RNA for 48 h, followed by erastin treatment; (E) Erastin+si‐AMPKα group: cells were transfected with si‐AMPKα for 48 h, followed by erastin treatment; (F) Erastin+si‐BECN1 group: cells were transfected with si‐BECN1 for 48 h, followed by erastin treatment; (G) Erastin+NAC group: cells were treated with N‐acetyl‐L‐cysteine (NAC; 500 μM; MCE, HY‐B0215, Shanghai, China) for 24 h, followed by erastin treatment; (H) Erastin+Mito‐TEMPO group: cells were treated with Mito‐TEMPO (50 μM; MCE, HY‐112879, Shanghai, China) for 24 h, followed by erastin treatment. In the final part, cells were divided into 6 groups: (A) Control group; (B) Erastin group; (C) Erastin+si‐NC group: cells were transfected with si‐RNA for 48 h, followed by erastin treatment; (D) Erastin+si‐ATG5 group: cells were transfected with si‐ATG5 for 48 h, followed by erastin treatment; (E) Erastin+si‐ATG7 group: cells were transfected with si‐ATG7 for 48 h, followed by erastin treatment; (F) Erastin+si‐NCOA4 group: cells transfected with si‐NCOA4 for 48 h, followed by erastin treatment.

### 2.7 Fe^2+^ Assay

Intracellular iron release was assessed using FeRhoNox‐1 (Goryo Chemical, GC901, Sapporo, Japan). Following cell treatment, the cells in 96‐well plates were incubated with FeRhoNox (5 μM) for 1 h, before being washed in PBS and incubated with HBSS (precell, PB180324, Wuhan, China) for 30 min. Fluorescence microscopy (Ex/Em = 540/575 nm) was employed for measurement. All assays were repeated in triplicate.

### 2.8 ROS Assay

The tissue and cells were processed into a single‐cell suspension using the medium, and the resulting cell suspension was inoculated into 12‐well plates with 1 mL cell suspension of 2 × 10^5^ cells per well. The plate was incubated overnight at 37°C in 5% CO_2_. According to the experimental group treatment, the cells were washed twice in PBS and then centrifuged at 1500 rpm for 5 min. Next, PBS was removed and the diluted 20 μM DHE (R001, Beijing, China) was added. The plate was incubated at 37°C for 30 min and shaken every 3 min. The cells were washed three times using a serum‐free medium. Finally, 500 μL of PBS was suspended and detected by flow cytometry. All assays were repeated five times.

### 2.9 Glutathione (GSH) Assay

GSH levels in cells and tissues were assessed using the GSH kit (Beyotime, S0053, Shanghai, China). Samples and standards were added, thoroughly mixed and 150 μL total glutathione detection solution was added. The mixture was then incubated at 25°C or room temperature for 5 min. Subsequently, 50 μL 0.5 mg/mL NADPH solution was added. After 25 min, the A412 samples were measured with a microplate reader, and the total glutathione was calculated. The content of GSSG was detected by removing GSH from samples with appropriate reagents. The GSH level was calculated by deducting the GSSG content from the total glutathione (GSSG+GSH). All assays were repeated five times.

### 2.10 Malondialdehyde (MDA) Assay

MDA content in cells and tissues was determined by the TBA method (Nanjing Jiancheng Institute of Bioengineering, A003‐4‐1, Nanjing, China). Following treatment, the cell culture supernatant was discarded and the cells were scraped off. A small portion of cells or tissues was taken for protein concentration detection. The kit extraction solution was added to the remaining cells or tissues. The cells or tissues were subjected to ultrasound disruption, forming a cell suspension. The working solution of the kit was added, and the mixture was placed in a water bath at 95°C for 40 min. After centrifugation, the supernatant was taken to measure the absorbance at 530 nm wavelength. Then, the MDA concentration was calculated according to the comparison with the test results of standard products. All assays were repeated five times.

### 2.11 Glutathione Peroxidase 4 (GPX4) Assay

Cell or tissue samples were first treated with lysis buffer (100 mM Tris, pH 7.6, 5 mM EDTA, 1 mM NaN3, and 0.1% oxide‐free Triton‐X100) before the lysate was supplemented with 0.6 U/mL glutathione reductase (Sigma, G3664, Shanghai, China), 0.2 mM NADPH (Sigma, N7505, Shanghai, China), 3 mM GSH (Sigma, G4251, Shanghai, China) and 200 mM CHP (Sigma, 247502, Shanghai, China). NADPH conversion rates were measured at 340 nm for 10 min at 37°C, and GPX4 enzyme activity was then calculated. All assays were repeated five times.

### 2.12 Cell Viability Assay

Cell viability was evaluated using the Cell Counting Kit‐8 (CCK‐8) (MCE, HY‐K0301, Shanghai, China), according to the manufacturer's instructions. Cells were seeded on a 96‐well flat bottom microtiter plate at a density of 5000 cells per well. After 24 h, the CCK‐8 agent was added at the indicated concentrations for 6 h. The absorbance was measured on a microplate reader (Molecular Devices Co., Sunnyvale, CA, USA) at 450 nm.

### 2.13 Lipid Peroxidation Assay

The levels of lipid peroxidation were detected using a C11‐BODIPY probe (Invitrogen, D3861, Shanghai, China). Cells were incubated with C11‐BODIPY (1 μM) in serum‐free medium for 30 min. Then, the cells were washed three times with PBS to remove excess C11‐BODIPY. The fluorescence of C11‐BODIPY was measured by flow cytometry (A00–1–1102, Beckman, USA).

### 2.14 Calcein‐AM/PI Staining Detection Level Detection

The cells were inoculated into 96‐well plates and treated with different drugs. Following treatment, the culture medium was removed, the cells were digested and washed with PBS. Then, 100 μL Calcein AM/PI (Calcein/PI Cell Activity and Cytotoxicity Assay Kit, Beyotime, C2015L, Shanghai, China) was added for the assay. The plate was incubated at 37°C for 30 min in the dark. After incubation for 1 h, the staining effect was observed under a fluorescence microscope (Olympus, IX51, Tokyo, Japan) (Calcein AM is green fluorescence, Ex/Em = 494/517 nm; PI is red fluorescence, Ex/Em = 535/617 nm). All assays were repeated in triplicate.

### 2.15 Autophagy Flow Detection

After the cells were grouped, the medium was replaced and 10 μL Ad‐Mcherry‐GDP‐LC3b virus stock solution (Biyuntian, C3011, Shanghai, China) was added to each well. After continuous culture at 37°C for 24 h, fresh culture‐medium without virus was added and the cells were washed with PBST. Autophagy flow was performed and photographed under a confocal laser fluorescence microscope (Olympus, FV3000, Tokyo, Japan) 48 h later. All assays were repeated in triplicate.

### 2.16 Transmission Electron Microscopy (TEM)

Following experimental treatments, HTR‐8/Svneo cells were fixed in 2.5% glutaraldehyde containing sodium cacodylate at 4°C for at least 6 h. The cells were next post‐fixed with 1% osmium tetroxide, dehydrated, embedded, and cut into 50 nm‐thick sections. After staining with 3% uranyl acetate and lead citrate, cellular ultrastructure was examined by TEM (FEI, Tecnai G20 TWIN, USA).

### 2.17 Western Blotting

RIPA lysate (Beyotime, P0013B, Shanghai, China) was used to lyse cells and tissues, and the sample was centrifuged at 4°C at 12000 rpm for 5 min. The supernatant was retained. BCA protein concentration assay kit (Beyotime, P0012S, Shanghai, China) was utilised to detect protein concentration. Then, 40 μg of each protein was mixed with loading buffer and the mixture was boiled for 5–10 min. After cooling and centrifugation, supernatant or Marker was added to the loading well. After sample addition, electrophoresis was performed at a constant pressure of 80 V until the bromophenol blue indicator formed a line at the junction of concentrated glue and separation glue, then the voltage was changed to a constant pressure of 120 V until bromophenol blue reached the bottom of the gel, which lasted approximately 1.5 h. The gel was removed from the electrophoresis chamber and placed in the electro‐transfer solution. PVDF membrane (Millipore, Billerica, MA, USA) was soaked in methanol for several seconds and then soaked in the electrotransfer buffer together with the filter paper. The black plate, fibre pad, filter paper, gel, PVDF membrane, filter paper, fibre pad and white plate were placed successively, and placed into the membrane transfer instrument after clamping. The protein transfer was performed at 300 mA for 1.5 h. Subsequently, the PVDF membrane was soaked in TBST containing 5% skim milk powder and blocked in a shaker at room temperature for 2 h. The corresponding primary antibody (GRα, Abcam, ab3580, 1: 1000, Hangzhou, China; FTH1, Affinity, DF6278, 1: 1000, Wuhan, China; GAPDH, Affinity, AF7021, 1:1000, Wuhan, China; AMPKα, Affinity, AF6423, 1:1000, Wuhan, China; p‐AMPKα, Affinity, AF3423, 1:1000, Wuhan, China; BECN1, Affinity, AF5128, 1:1000, Wuhan, China; p‐BECN1 (S90), CST, 864555, 1:1000, Wuhan, China; p‐BECN1 (S93), CST, 14717, 1:1000, Wuhan, China; ATG5, Affinity, DF6010, 1:1000, Wuhan, China; ATG7, Affinity, DF6130, 1:1000, Wuhan, China; NCOA4, Affinity, DF4255, 1:1000, Wuhan, China; LC3, Affinity, AF5402, 1:1000, Wuhan, China; p62, Affinity, AF5384, 1:1000, Wuhan, China; GAPDH, Affinity, AF7021, 1:1000, Wuhan, China) were used. PVDF membrane was soaked in primary antibody incubation solution and incubated at 4°C overnight. The next day, the PVDF membrane was washed 5–6 times for 5 min each using TBST. Next, the secondary antibody (Goat Anti‐Rabbit IgG (H+L) HRP, affinity, S0001, 1:5000, Wuhan, China) was diluted in the blocking solution, applied to the PVDF membrane and incubated in a shaker at 37°C for 2 h. Again, TBST was used to fully wash PVDF membrane 5–6 times, 5 min/time. The ECL reagent (Millipore, Billerica, MA, USA) and the stable peroxidase solution were mixed in a ratio of 1:1, and the working solution was added to the PVDF membrane by drops. After the reaction proceeded for a few minutes, the excess substrate solution was absorbed by filter paper after the fluorescence band was obvious. Western blot results were analysed using ImageJ software (National Institutes of Health, Bethesda, USA). All assays were repeated in triplicate.

### 2.18 Data Analysis

Statistical software Prism 9.0 was used for data analysis, and all data were expressed as Mean ± SD. The comparison between two groups was conducted using the student *t*‐test, while the comparison among more than two groups was conducted using one‐way ANOVA. *p* < 0.05 was considered statistically significant.

## 3 Results

### 3.1 Prenatal Administration of DEX Led to Significantly Enhanced Placental Ferroptosis Activity

**FIGURE 1:**
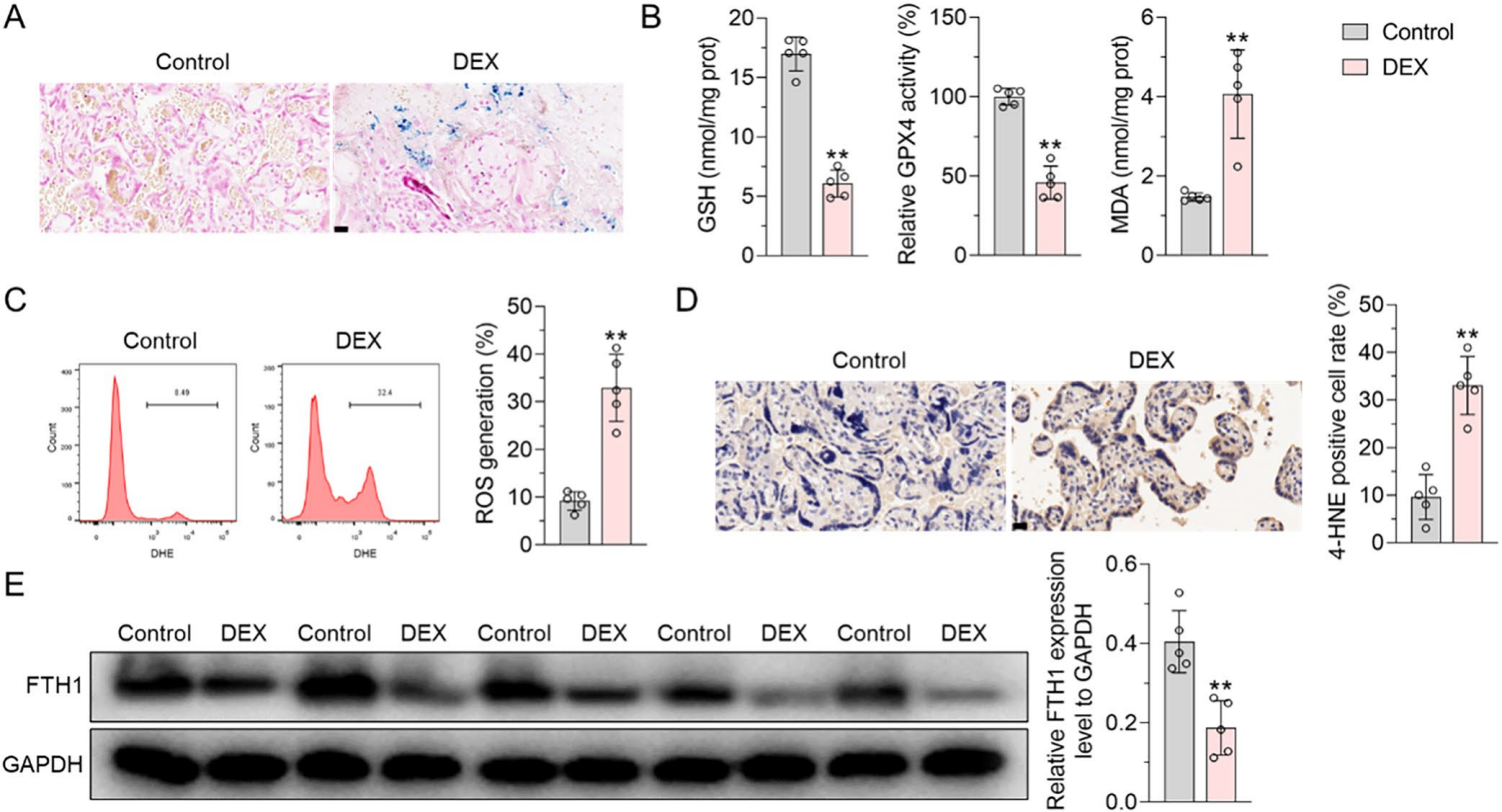
DEX treatment increased ferroptosis activity in placental tissues. (A) Representative images of Prussian Blue staining of placental tissues. (B) The expression level of GSH, GPX4 and MDA as determined by a reagent kit. (C) Measurement of ROS level was measured by flow cytometry. (D) Immunohistochemistry staining of 4‐HNE, and the number of 4‐HNE‐positive cells in each region. (E) The protein expression of FTH1 was determined by western blot. Data shown in the bar chart are Mean ± SD, statistical comparisons between the two groups were performed using the student *t*‐test. ** represent *p* < 0.01 VS. The scales in (A) and (D) were 20 μm.

Prussian blue staining was employed to explore the localization of iron deposition in placental tissues between the control group and the DEX group. The results indicated excessive accumulation of iron in the placental tissues from the DEX group compared to the control group (Figure 1A). Ferroptosis, characterised by heightened lipid peroxidation mediated by Fe(II) [32], was further investigated. Our results demonstrate a decrease in the contact of GSH and the activity of GPX4 in placental tissue of DEX compared to the control group. Concurrently, the content of MDA, ROS and the expression level of lipid peroxidation biomarker 4‐HNE were increased (Figure 1B–D). Moreover, we further examined the protein levels associated with ferritin, such as FTH1. Western blot analysis demonstrated that the expression level of FTH1 protein in the DEX group was significantly lower than that in the control group (Figure 1E). Consequently, the clinical observation in this study implies that DEX may influence the ferroptosis level of placental cells, while the specific mechanism needs further clarification.

### 3.2 Ferrostatin 1 or DFOM, the Ferroptosis Inhibitor, Reverses the Effect of DEX on the Ferroptosis Level of Placental Trophoblast Cells

**FIGURE 2:**
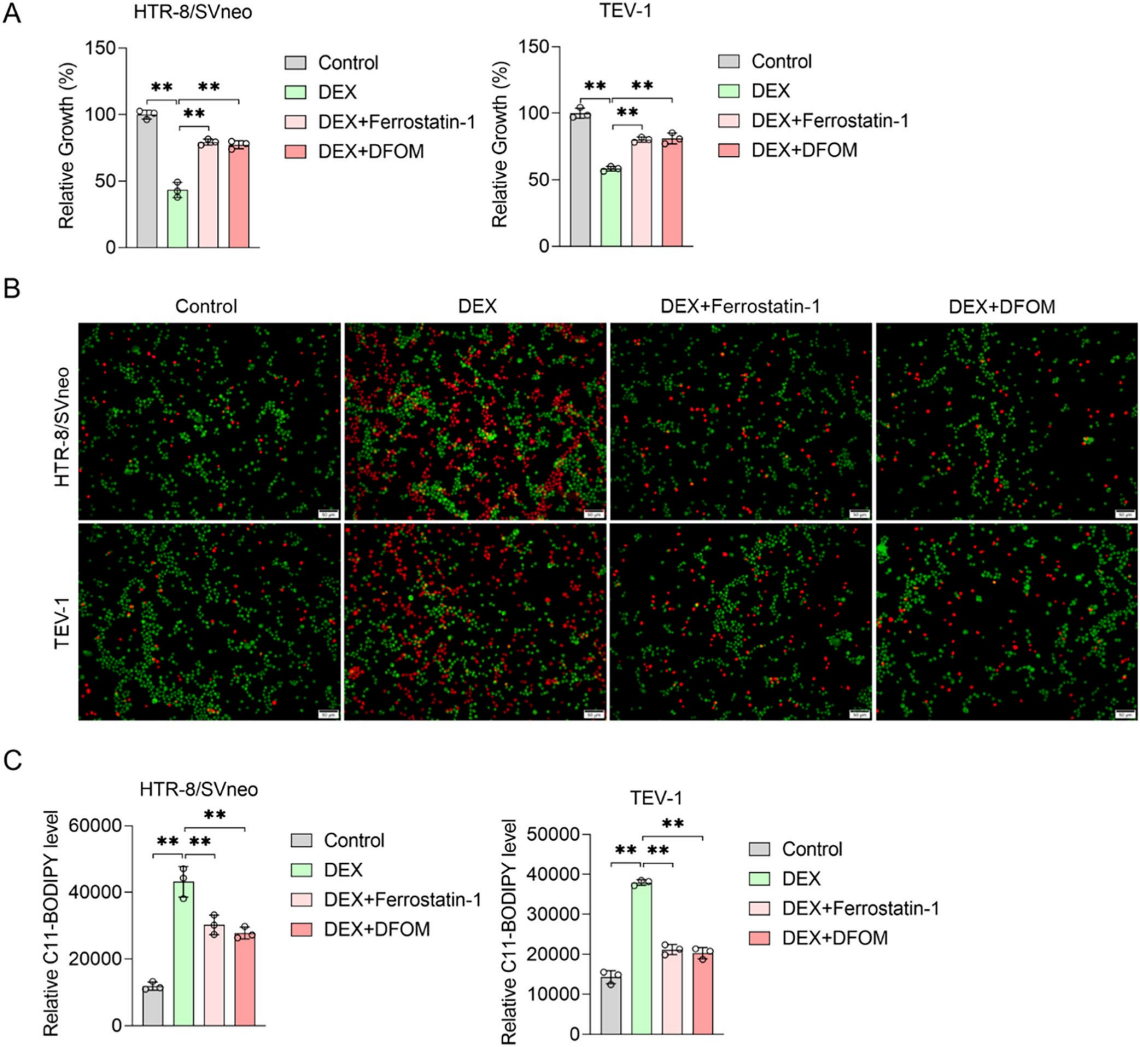
DEX promoted ferroptosis of HTR8/SVeno and TEV‐1. (A) The viability of cells was quantified via the CCK8 assay. (B) Live and dead cells were detected by Calcein‐AM/PI double staining. Green represents calcein‐positive cells (living cells), whereas red represent PI‐positive cells (dead cells). (C) Lipid peroxidation was measured by the C11‐BODIPY assay. Data shown in the bar chart are presented as Mean ± SD. Inter‐group statistical analysis was performed by one‐way ANOVA. ** represent *p* < 0.01, and the scale in (B) was 50 μm.

Building upon the outcomes of the aforementioned clinical trials, we next investigated in depth the effect of DEX on trophoblast ferroptosis. Two human trophoblast cell lines were employed in this study: HTR‐8/SVneo and TEV‐1. First, each trophoblast cell line was treated with DEX for 72 h before the CCK‐8 assay was performed to assess cell viability. The results demonstrated a significant decrease in the viability of HTR‐8/SVneo and TEV‐1 cells after DEX addition, compared to the control group. Notably, the ferroptosis inhibitors Ferrostatin‐1 and DFOM could partially save cell viability (Figure 2A). To distinguish between living and dead cells, Calcein‐AM/PI staining was utilised. Calcein‐labelled living cells emitted a green fluorescence, and PI‐labelled dead nuclei emitted a red fluorescence. Under DEX conditions, the number of living cells decreased significantly in both HTR‐8/SVneo and TEV‐1 cells while the number of dead cells increased under DEX treatment. In contrast, supplementation of Ferrostatin‐1 and DFOM decreased the number of dead cells (Figure 2B). Considering lipid peroxidation is an insufficient but necessary event during ferroptosis [33], we next evaluated lipid peroxidation levels and antioxidant activity after DEX as indicators of ferroptosis. Results indicated that the addition of Ferrostatin‐1 and DFOM inhibited the content of lipid peroxide in cells after DEX treatment (Figure 2C). Therefore, our findings suggest that DEX inhibited the viability and proliferation of HTR‐8/SVneo and TEV‐1 cells in vitro in a ferroptosis‐dependent manner.

### 3.3 DEX Promotes Ferroptosis of Placental Trophoblast Cells by Tuning GRα

**FIGURE 3:**
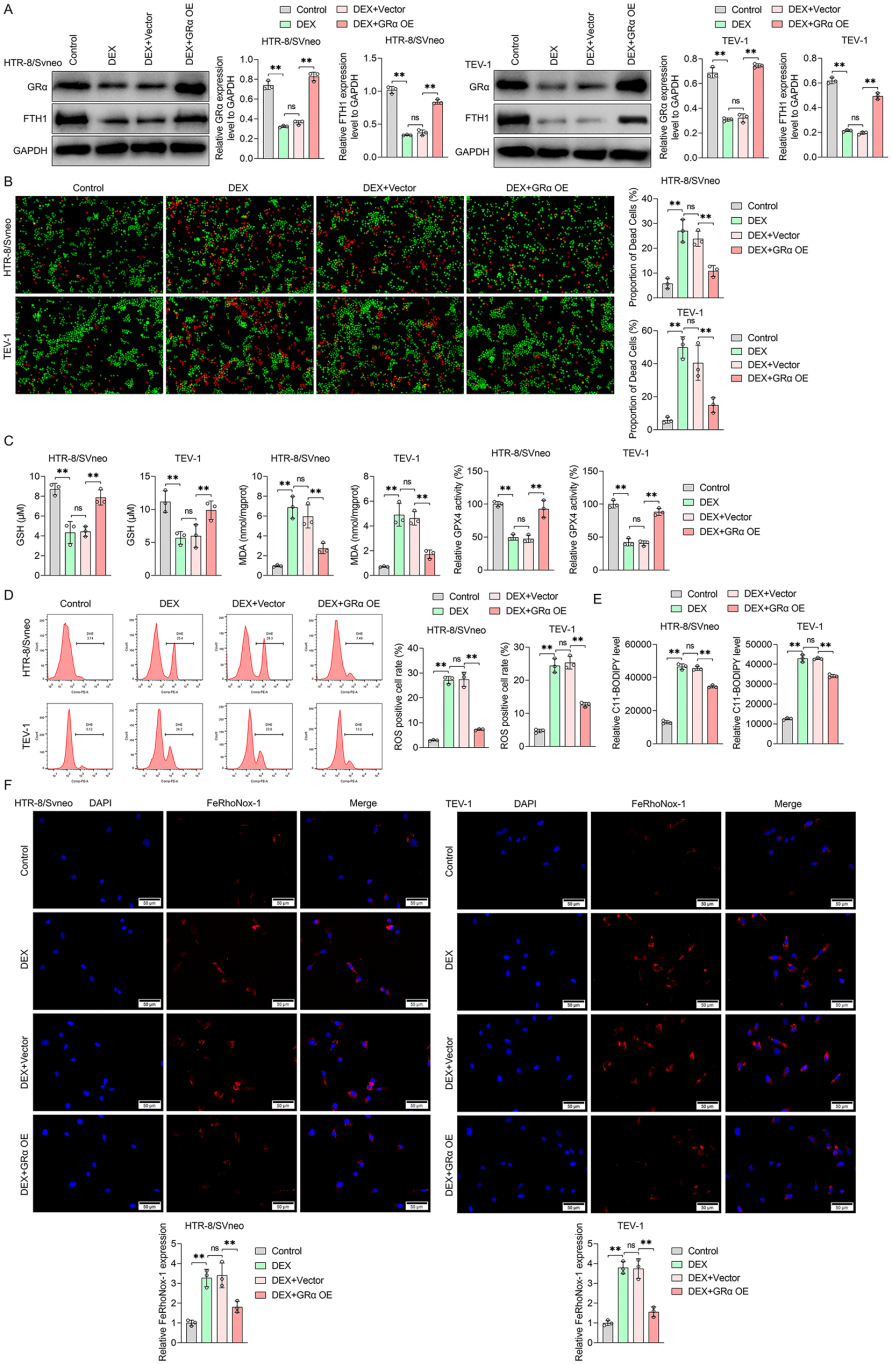
DEX promoted ferroptosis of HTR8/SVeno and TEV‐1 cells via GRα. (A) The protein levels of GRα and FTH1 were measured by western blot. (B) Representative images of Calcein‐AM/PI double staining showing live and dead cells. (C) The expression level of GSH, MDA, and GPX4 proteins. (D) Measurement of ROS production by flow cytometry. (E) Lipid peroxidation was detected by the C11‐BODIPY assay. (F) The release of Fe^2+^ was examined by FeRhoNox‐1. Data shown in the bar chart are presented as Mean ± SD, inter‐group statistical comparisons were performed by one‐way ANOVA. Ns represent *p* > 0.05, and ** represent *p* < 0.01. The scales in (B) and (F) were 50 μm.

Given that intracellular glucocorticoid action is regulated by glucocorticoid receptors (GR) [34], next, we investigated the expression of Grα (one of the major variants of GR expressed in the placenta) in placental trophoblast cells HTR‐8/SVneo and TEV‐1 cells. Our findings indicated that DEX decreased the expression of GRα protein in placental trophoblast cells (Figure 3A). However, the involvement of GRα in the regulation of DEX‐induced ferroptosis in placental trophoblast cells is unclear. To explore this, we transfected HTR‐8/SVneo and TEV‐1 cells with an overexpressed lentivirus (Grα OE) to upregulate GRα. We examined the validation of GRα overexpression in HTR‐8/SVneo cells (Figure S1). Subsequently, placental trophoblast cells were treated with DEX after transfection of Grα OE lentivirus to investigate whether DEX acts on GRα to regulate ferroptosis. The results revealed that DEX treatment increased cell death in placental trophoblast cells, and this effect was counteracted by GRα OE (Figure 3B). Additionally, FTH1 protein expression was increased in the DEX and GRα OE group compared with the DEX treatment group (Figure 3A). Importantly, intervention by GRα OE in placental trophoblast cells reversed DEX‐induced reductions in GSH and GPX4 and increased MDA levels (Figure 3C). Compared with the DEX treatment group, ROS and lipid oxidation levels were significantly decreased after transfection with GRα OE, and the fluorescence intensity of Fe^2+^ was significantly decreased (Figure 3D–F). These results suggest that GRα OE has the potential to reverse DEX‐induced ferroptosis in HTR‐8/SVneo and TEV‐1 cells.

### 3.4 DEX Promotes Autophagy of HTR‐8/Svneo Cells by Tuning GRα

**FIGURE 4:**
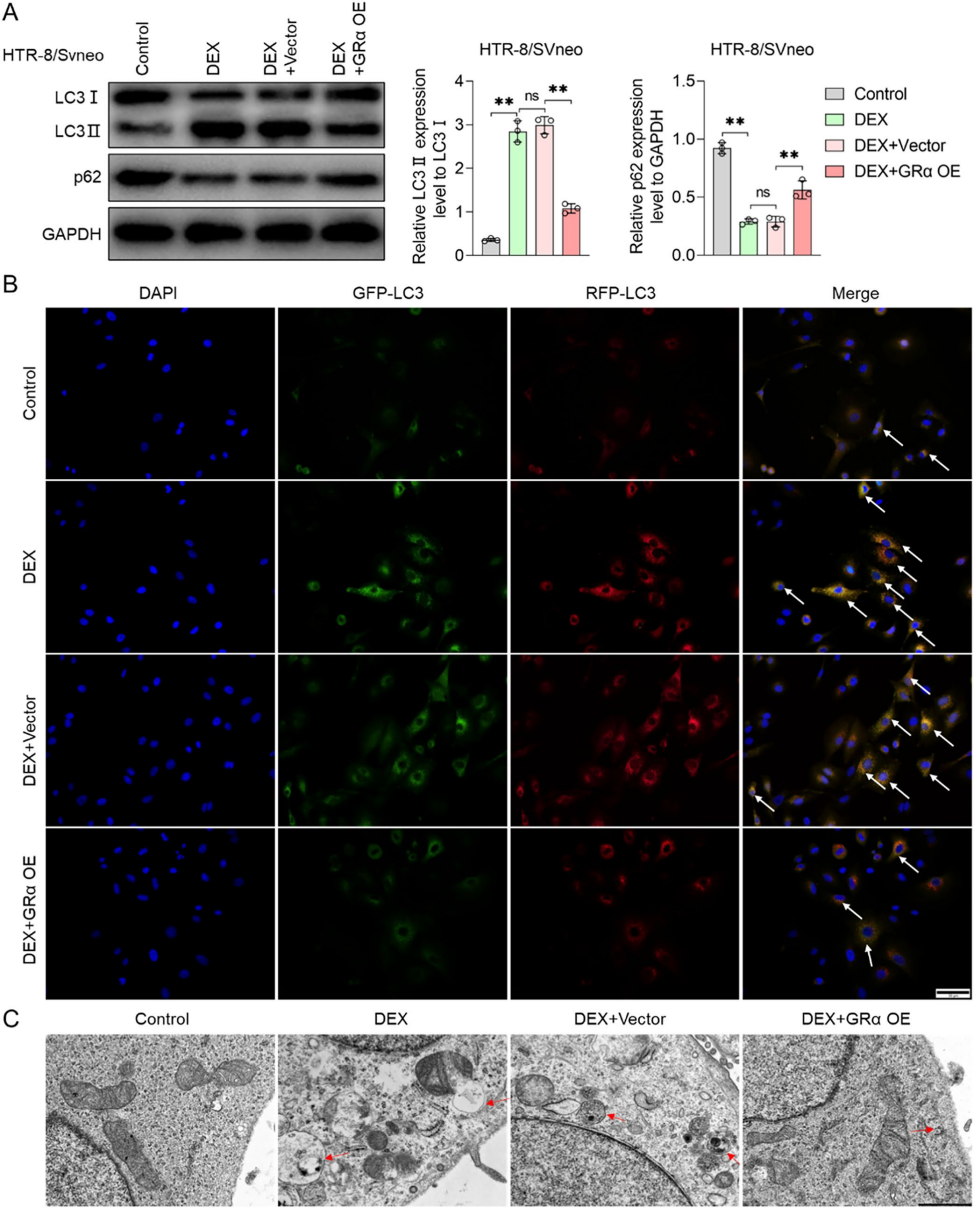
DEX enhanced autophagy of HTR8/SVeno cells via GRα. (A) The protein levels of LC3‐I/II and p62 were measured by western blot. (B) Fluorescence images of cells expressing mCherry‐GFP‐LC3B. (C) TEM images of cells, with red arrows indicating the autophagosome. Data shown in the bar chart are presented as mean ± SD, inter‐group statistical analyses were performed by one‐way ANOVA. Ns represent *p* > 0.05, and ** represent *p* < 0.01. The scales in (B) and (C) were 50 and 1.0 μm, respectively.

Subsequently, we transfected Grα OE lentivirus into HTR‐8/Svneo cells and subjected the cells to DEX to determine whether DEX acts on GRα to regulate autophagy. Relative to the control group, the protein levels of LC3‐II were increased in the DEX group, alongside a decrease in the protein levels of LC3‐I and p62. The effect of DEX on LC3‐II was eliminated by GRα OE (Figure 4A). Likewise, GRα OE reversed the DEX‐induced increase in the number of GFP‐LC3 spots (Figure 4B). These findings indicated that DEX promoted autophagosome formation in HTR‐8/Svneo cells by inhibiting GRα. In addition, utilising the transmission electron microscopy (TEM), the gold standard for autophagy detection, we found an increased presence of autophagosomes in HTR‐8/Svneo cells with DEX and a corresponding decrease in the number of autophagosomes in GRα OE cells (Figure 4C). In conclusion, these results suggested that DEX enhanced autophagy in HTR‐8/Svneo cells by inhibiting the expression of GRα.

### 3.5 DEX Activated AMPKα/BECN1 and ATG5/ATG7/NCOA4 Signalling Pathways by Inhibiting GRα

**FIGURE 5:**
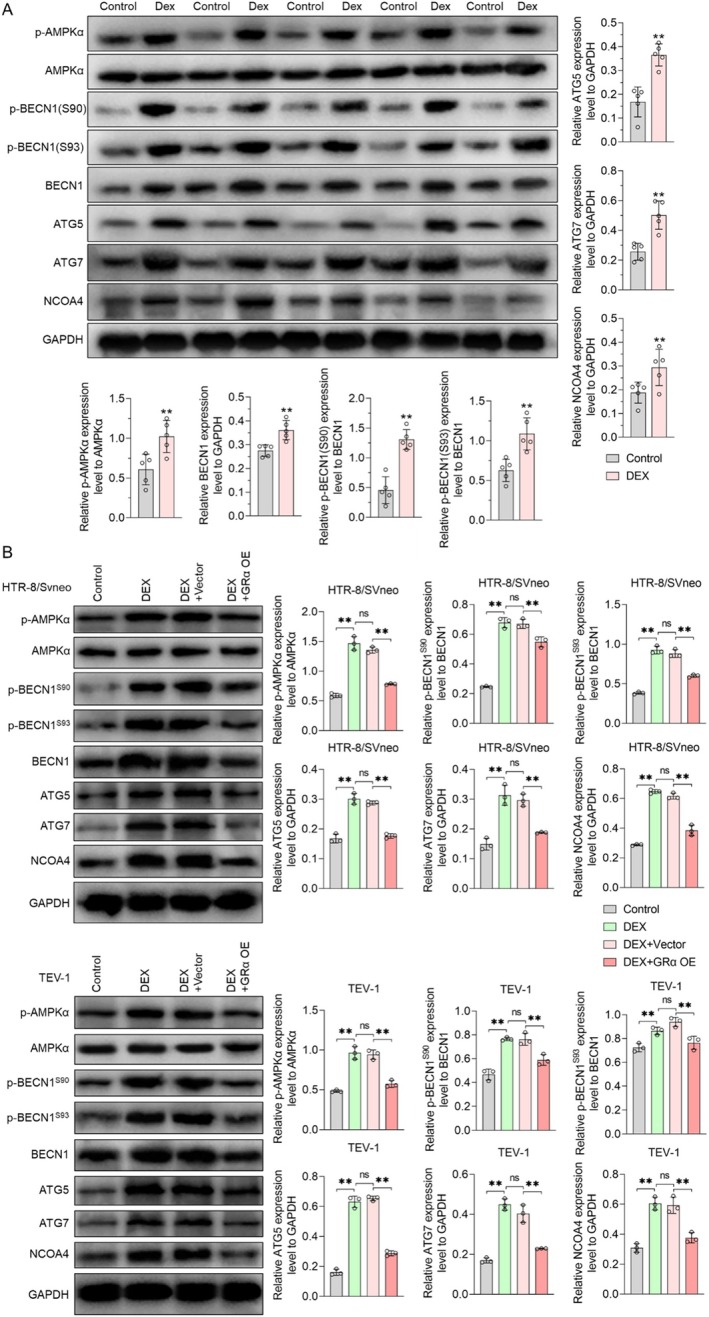
DEX activated the AMPKα/BECN1 and ATG5/ATG7/NCOA4 signalling pathways via GRα. (A) Representative western blot results of placental tissues. (B) Representative western blot results showing HTR8/SVeno and TEV‐1 expression. Data shown in the bar chart are presented as mean ± SD. Statistical analysis between two groups in (A) was performed by the student *t*‐test, inter‐group statistical analysis in (B) was performed by one‐way ANOVA. ** in (A) represent *p* < 0.01 VS. Control. Ns represent *p* > 0.05, and ** represent *p* < 0.01 in (B).

To ascertain the potential impact of DEX on AMPKα/BECN1 and ATG5/ATG7/NCOA4 as potential pathways, we measured the total levels of AMPKα, BECN1, ATG5, ATG7, NCOA4 and the phosphorylation levels of AMPKα and BECN1 on clinical placental tissue samples. Compared with the control group, the protein expression levels of p‐AMPKα, BECN1, p‐BECN1 (S90), p‐BECN1 (S93), ATG5, ATG7 and NCOA4 increased, while the total AMPKα protein was unchanged (Figure 5A). This suggested that DEX intervention might activate both AMPKα/BECN1 and ATG5/ATG7/NCOA4 signalling pathways. Next, we transfected Grα OE into HTR‐8/SVneo and TEV‐1 cells to further explore the relationship between DEX, GRα, AMPKα/BECN1 and ATG5/ATG7/NCOA4. We observed that the effects of DEX on the total levels of AMPKα, BECN1, ATG5, ATG7, NCOA4 and the phosphorylation levels of AMPKα and BECN1 in cells were consistent with the results of clinical samples. The effects of DEX on AMPKα/BECN1 and ATG5/ATG7/NCOA4 were nullified by GRα OE (Figure 5B). These results indicated that DEX regulated AMPKα/BECN1 and ATG5/ATG7/NCOA4 signalling pathways through GRα.

### 3.6 DEX Promotes Autophagy of Placental Trophoblast Cells by Activating AMPKα/BECN1 and ATG5/ATG7/NCOA4 Signalling Pathways

**FIGURE 6:**
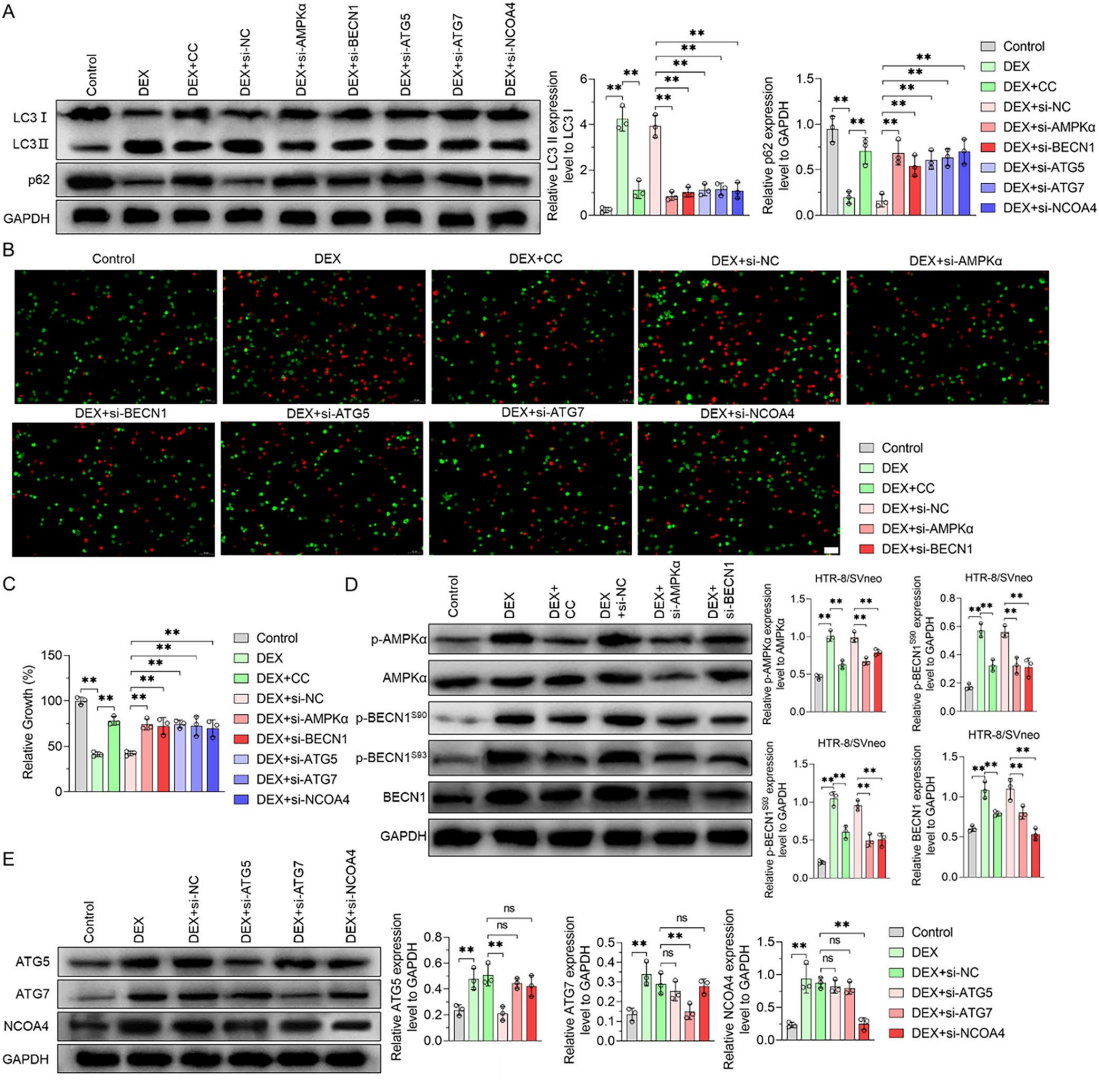
DEX promoted autophagy in HTR8/Sveno cells by activating AMPKα/BECN1 and ATG5/ATG7/NCOA4 signalling pathways. (A) Representative western blot results; (B) Representative immunofluorescence images of Calcein‐AM/PI double staining. (C) Cell viability was examined by the CCK8 assay. (D–E) Representative western blot results. Data shown in the bar chart are presented as the mean ± SD. Inter‐group statistical analysis was performed by one‐way ANOVA. Ns represent *p* > 0.05, and ** represent *p* < 0.01. The scale in (B) was 50 μm.

The AMPKα/BECN1 and ATG5/ATG7/NCOA4 signalling pathways play pivotal roles in regulating autophagy. To investigate whether DEX promotes autophagy in HTR‐8/SVneo cells through these signalling pathways, we used CC (an AMPK inhibitor) and siAMPKα to block the expression of AMPKα in HTR‐8/SVneo cells, and siBECN1, siATG5, siATG7 and siNCOA4 to block the expression of BECN1, ATG5, ATG7 and NCOA4, respectively. The results shown in Figure S2 verified that siRNA reduced the expression of AMPKα, BECN1, ATG5, ATG7 and NCOA4. The results demonstrated that CC, siAMPKα, siBECN1, siATG5, siATG7 and siNCOA4 reversed the promoting effect of DEX on the ratio of LC3 II/I and the inhibiting effect of p62 protein expression (Figure 6A). Additionally, results from Calcein‐AM/PI staining and CCK8 analysis showed that the influence of DEX on HTR‐8/SVneo cells was eliminated by CC, siAMPKα, siBECN1, siATG5, siATG7 and siNCOA4 (Figure 6B,C). Therefore, CC, siAMPKα, siBECN1, siATG5, siATG7 and siNCOA4 inhibited the autophagy promotion of HTR‐8/SVneo cells induced by DEX.

We then assessed the effects of CC, siAMPKα, and siBECN1 on the expression levels of total AMPKα, BECN1, and phosphorylated AMPKα and BECN1, and the effects of siATG5, siATG7 and siNCOA4 on the expression levels of ATG5, ATG7 and NCOA4. Treatment of cells with CC or siAMPKα resulted in a decline in the expression levels of p‐AMPKα in HTR‐8/SVneo, leading to a concomitant decrease in the levels of BECN1, p‐BECN1 (S90) and p‐BECN1 (S93). Conversely, treatment with siBECN1 led to a reduction in BECN1 and p‐BECN1 (S90 and S93) levels, but p‐AMPKα levels were not affected (Figure 6D). Similarly, after siATG5 treatment of HTR‐8/SVneo cells, ATG5 levels decreased, but the expression of ATG7 and NCOA4 was not affected. Treatment with siATG7 reduced the ATG7 level, while the ATG5 and NCOA4 levels remained unchanged. Similarly, si‐NCOA4 decreased NCOA4 levels without affecting the levels of ATG5 and ATG7 (Figure 6E). These results indicated that DEX promoted autophagy of placental trophoblast cells by activating AMPKα/BECN1 and ATG5/ATG7/NCOA4 signalling pathways.

### 3.7 Blocking the AMPKα/BECN1 or ATG5/ATG7/NCOA4 Signalling Pathways Attenuates Erastin‐Induced Ferroptosis of Placental Trophoblast Cells

**FIGURE 7:**
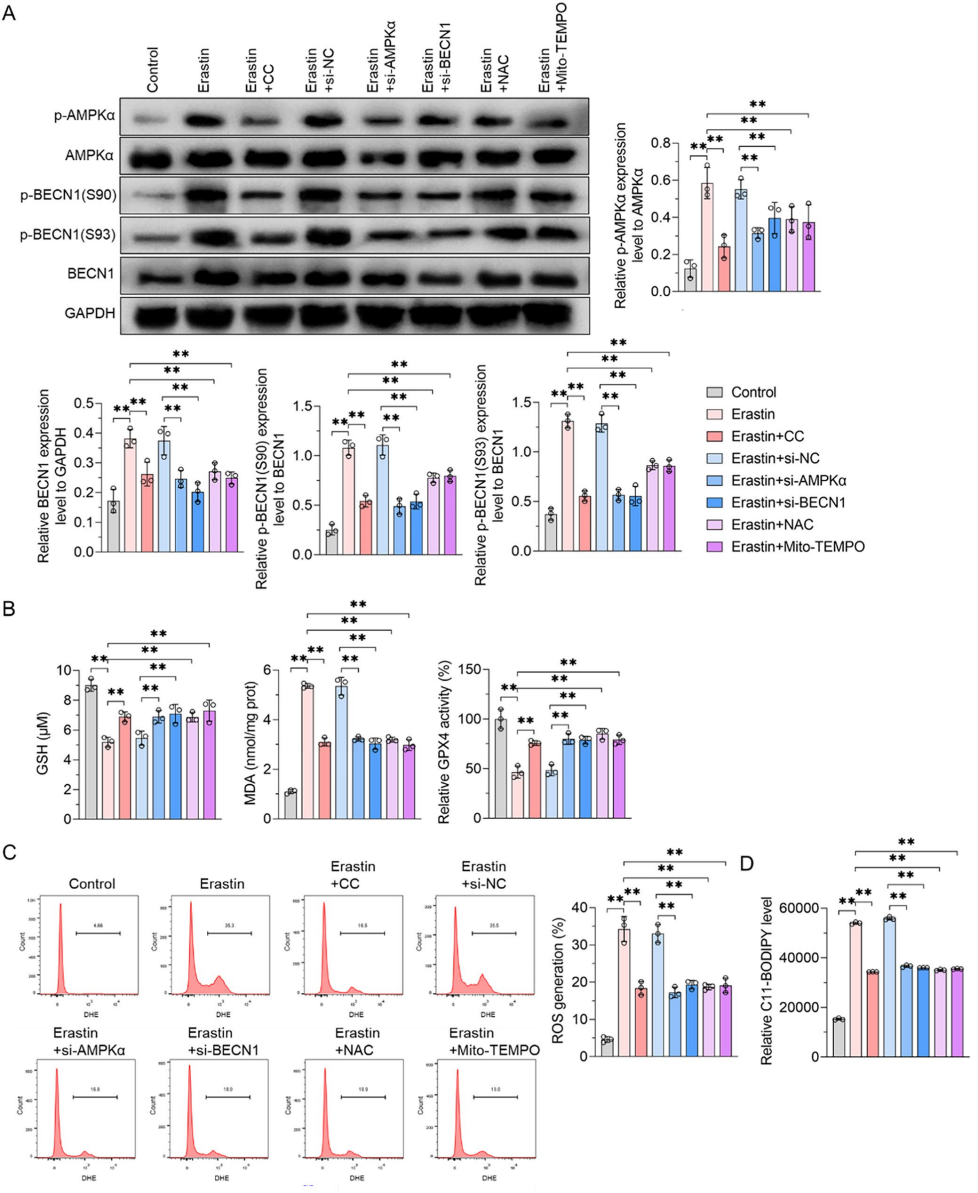
Inhibition of the AMPKα/BECN1 signalling pathway can inhibit erastin‐induced ferroptosis of placental trophoblast cells. (A) Representative western blot results. (B) The expression level of GSH, GPX4, and MDA proteins were measured by the respective reagent kit. (C) ROS production was measured by flow cytometry. (D) Lipid peroxidation was measured by the C11‐BODIPY assay. Data shown in the bar chart are presented by Mean ± SD. Inter‐group statistical analysis was performed via one‐way ANOVA. ** represent *p* < 0.01.

**FIGURE 8:**
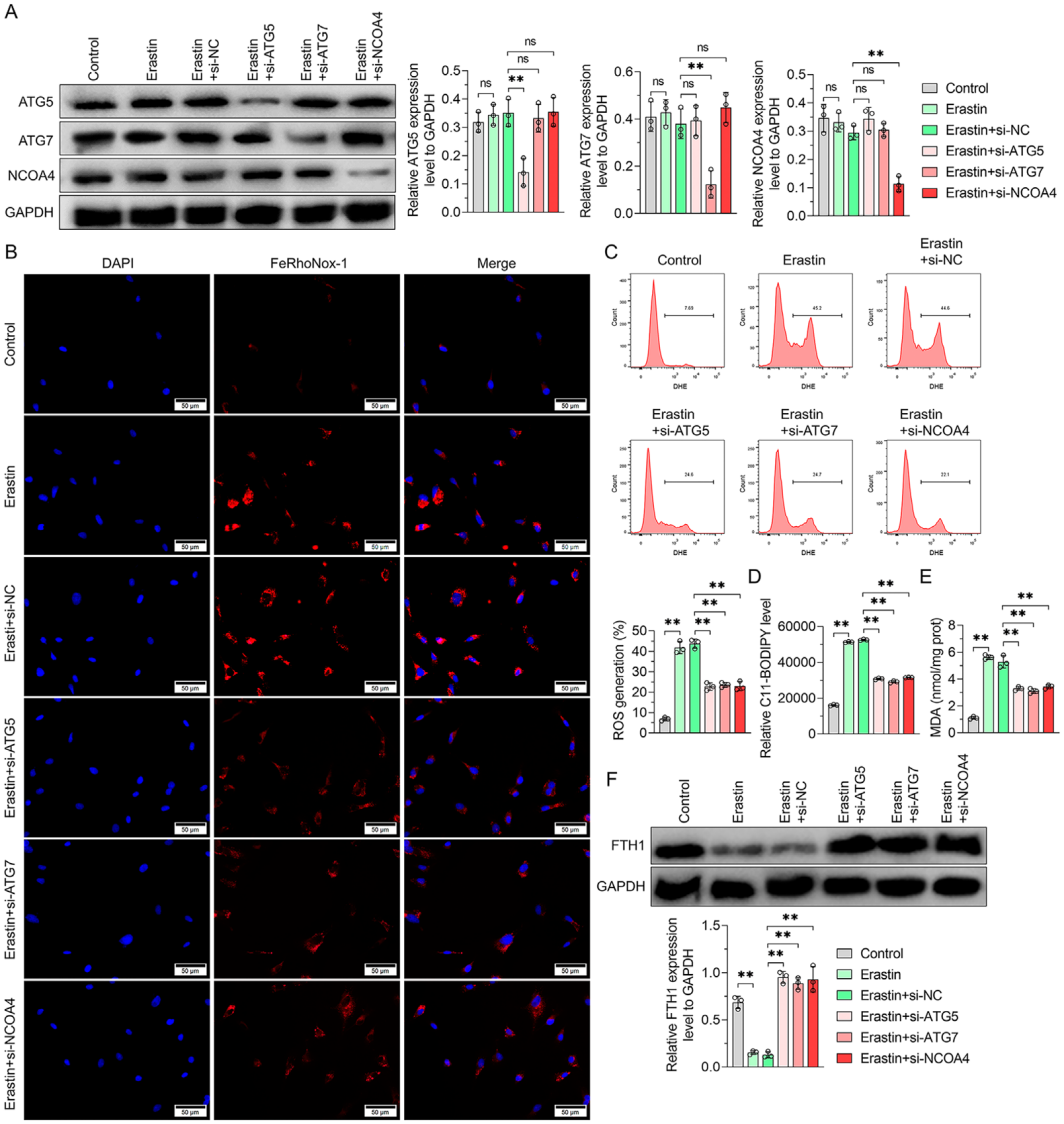
Inhibition of the ATG5/ATG7/NCOA4 signalling pathway may mitigate erastin‐induced ferroptosis of placental trophoblast cells. (A) Representative western blot results. (B) Measurement of Fe^2+^ release by the FeRhoNox‐1 assay. (C) ROS production was detected by flow cytometry. (D) Lipid peroxidation was examined by the C11‐BODIPY assay. (E) The expression of MDA was measured by a commercial reagent kit. (F) The expression levels of GRα and FTH1 proteins were quantified by western blot. Data shown in the bar chart presented as the mean ± SD. Inter‐group statistical analysis was performed by one‐way ANOVA. Ns represent *p* > 0.05, and ** represent *p* < 0.01. The scale in (B) was 50 μm.

Ferroptosis is frequently accompanied by autophagy and is regarded as an autophagy‐dependent mode of cell death. We explored whether AMPKα/BECN1 and ATG5/ATG7/NCOA4 signalling pathways could regulate ferroptosis in HTR‐8/SVneo cells. Treatment with Erastin, a classical inducer of ferroptosis, in HTR‐8/SVneo cells, resulted in increased levels of p‐AMPKα, leading to elevated levels of BECN1, p‐BECN1 (S90) and p‐BECN1 (S93). Mito‐TEMPO, a mitochondrial‐targeted superoxide dismutase mimic, is commonly used as a mitochondrial ROS scavenger. The experimental results of GSH, MDA, GPX4, ROS and lipid oxidation levels showed that CC, siAMPKα, siBECN1, N‐acetylcysteine (NAC) and Mito‐TEMPO attenuated erastin‐induced ferroptosis and attenuated the activation of the AMPKα/BECN1 pathway (Figure 7A–D). Moreover, erastin did not increase the expression levels of ATG5, ATG7 and NCOA4 in HTR‐8/SVneo cells (Figure 8A). However, intracellular Fe^2+^, ROS, lipid oxidation levels and end products of lipid peroxidation (e.g., MDA) were significantly reduced in siATG5 or siATG7 cells after erastin treatment (Figure 8B–E), suggesting that ATG5 and ATG7‐mediated autophagy contributed to ferroptosis. Autophagy‐dependent ferroptosis, commonly known as ferritin autophagy, involves the autophagic degradation of ferritin (such as FTH1). Abnormal ferritin phagocytosis may contribute to intracellular iron accumulation and oxidative damage through the Fenton reaction [35]. Notably, protein levels of FTH1 were significantly increased in siATG5 and siATG7 cells subjected to erastin treatment (Figure 8F), suggesting that ATG5 and ATG7‐mediated autophagy was necessary for ferritin degradation. Although NCOA4 protein levels did not change significantly under erastin treatment, siNCOA4 increased FTH1 expression in HTR‐8/SVneo cells (Figure 8F). These findings suggested that AMPKα/BECN1 and ATG5/ATG7/NCOA4 signalling pathways are involved in autophagy‐dependent ferroptosis in HTR‐8/SVneo cells.

## 4 Discussion

DEX, a synthetic glucocorticoid, has been extensively utilised to treat preterm birth‐related pregnancy disorders and has been the focus of many clinical studies and animal experiments owing to the adverse effects of prenatal exposure to synthetic glucocorticoids on foetal development [4]. The placenta is the barrier between mother and fetus, and inadequate invasion of trophoblast cells stands as a primary contributor to placental dysfunction [36]. A previous study in our laboratory highlighted that early DEX treatment induced apoptosis in sheep placental cells [7]. Ferroptosis, a novel form of cell death different from apoptosis, is mainly attributed to abnormal oxidation disturbance of the intracellular microenvironment [9]. Existing literature suggests that ferroptosis of trophoblast cells plays a role in placental dysfunction and participates in the occurrence and development of placenta‐related diseases such as pre‐eclampsia, foetal growth restriction, premature delivery and abortion [37]. However, the question of whether DEX induces placental trophoblast cell death through the ferroptosis pathway remains unanswered.

In this context, the analysis of clinical samples from placental tissues revealed a notable increase in cell death, coupled with a decrease in GSH content and GPX4 activity, and an elevation in MDA and ROS levels, alongside heightened expression of the lipid peroxidation biomarker 4‐HNE. These changes align with the typical characteristics of ferroptosis [11], in the placenta subjected to prenatal DEX treatment. In addition, DEX reduced HTR‐8/SVneo and TEV‐1 cell viability and enhanced lipid oxidation fluorescence intensity. These changes were mitigated by Ferrostatin‐1 and DFOM treatments with ferroptosis inhibitors. Therefore, DEX can induce ferroptosis in placental trophoblast cells HTR‐8/SVneo and TEV‐1.

Glucocorticoids exert their biological role primarily through glucocorticoid receptors (GRs), comprising two main subunits (GRα and GRβ). GRα is recognised as the main mediator of glucocorticoid action, regulating gene clusters, particularly in the context of endogenous glucocorticoids [38]. Previous studies have highlighted that DEX can elevate the level of ROS in human placental JEG‐3 cells by down‐regulating GRα, and activating the ROS/AMPK pathway [14]. Hence, this study focused on GRα. As indicated, DEX led to the down‐expression of GRα protein in HTR‐8/SVneo and TEV‐1. The up‐regulation of GRα in HTR‐8/SVneo and TEV‐1 cells increased the content of GSH and GPX4 and decreased the levels of MDA, ROS, lipid ROS and Fe^2+^, suggesting that overexpression of GRα can reverse the DEX‐induced ferroptosis. Therefore, it is reasonable to infer that DEX induces ferroptosis of HTR‐8/SVneo and TEV‐1 cells by inhibiting GRα.

Recent studies have demonstrated ferroptosis as a form of autophagic cell death [39]. Autophagy plays a key role in governing ferroptosis by overseeing cellular iron homeostasis and cell ROS production [40]. Central to the mediation of autophagy are autophagy associated (ATG) genes. Among them, ATG5 and ATG7 are crucial for the formation of autophagosomes [35]. In this study, DEX significantly increased LC3‐II, autophagosome accumulation, and ATG5 and ATG7 protein expressions, underscoring its role as a key positive regulator of autophagy. This is noteworthy in the context of autophagy‐dependent ferroptosis, also known as ferritin autophagy—a process of autophagic degradation of ferritin (such as FTH1). Dysregulated ferritin phagocytosis causes intracellular iron accumulation and oxidative damage through Fenton reaction [35]. In the present study, we found that the expression of FTH1 protein was downregulated in the placenta of the prenatal DEX treatment group, and that DEX reduced protein expression of FTH1 in HTR‐8/SVneo and TEV‐1 cells by inhibiting GRα. Furthermore, siATG5 and siATG7 significantly increased protein levels of FTH1 in erastin treatment cells and erastin‐induced ferroptosis events were partially ablated by these interventions. These results indicate that autophagy mediated by ATG5 and ATG7 is necessary for ferritin degradation. NCOA4 mediated ferritin autophagy degradation is a key process of ferritin autophagy. NCOA4 can specifically recognise and degrade ferritin and increase intracellular iron levels, further contributing to oxidative damage caused by Fenton reaction. Likewise, siNCOA4 significantly increased FTH1 protein levels in erastin treatment cells, concurrently partially mitigating erastin‐induced ferroptosis events. It is suggested that NCOA4‐mediated ferritin degradation was related to ferroptosis. Thus, the results suggested that DEX induces autophagy‐dependent ferroptosis in HTR‐8/SVneo and TEV‐1 cells by inhibiting GRα.

AMPKα, functioning as a pivotal regulator of cell survival or death, holds a crucial role in orchestrating cellular defences against hypoxia, ischaemia and oxidative stress. Notably, the AMPK pathway is a common thread linking the mechanisms of ferroptosis and autophagy [41]. AMPKα‐mediated BECN1 phosphorylation at S90 and S93 not only promotes autophagy and cell transport, but also induces ferroptosis [42]. We hypothesised that DEX activates the AMPKα–BECN1 pathway, thereby inducing autophagy‐dependent ferroptosis in HTR‐8/SVneo cells. Consistent with our hypothesis, this study demonstrated that DEX up‐regulated AMPKα phosphorylation and increased BECN1 activity in both placental tissue samples and placental trophoblast cells. Thus, the involvement of AMPK in the mechanism of the effect of DEX on autophagywas confirmed in this study. CC partially blocked p‐AMPKα protein levels under DEX induction, accompanied by LC3‐II transformation and a reduction in the protein levels of BECN1, p‐BECN1 (S90) and p‐BECN1 (S93). Thus, this suggested that DEX instigates the induction of autophagy via the activation of AMPKα‐BECN1 pathway. This activation of autophagy, in turn, led to lipid peroxidation and iron accumulation as part of the response to the induction of ferroptosis. Our data strongly supported the notion that erastin‐induced ferroptosis was ameliorated by CC, siAMPKα, siBECN1, NAC and Mito‐TEMPO in HTR‐8/SVneo cells.

## 5 Conclusions

In this investigation, DEX, a prominent glucocorticoid employed in the treatment of preterm birth‐related pregnancy diseases, was employed to examine potential adverse effects of DEX on the placenta in women at risk of preterm birth. The study found that DEX causes the inhibition of placental trophoblast cell viability, attributed to ferroptosis induced by DEX within placental tissue. In conclusion, the results suggested that DEX inhibits GRα and increases ferroptosis in placental trophoblast cells. This phenomenon is intricately linked to the activation of autophagy, which assumes a death‐promoting role in cell death. Particularly noteworthy is ferritin autophagy, involving the degradation of FTH1, leading to iron overload, excessive lipid peroxidation and ultimately ferroptosis in response to DEX exposure. Additionally, the coordinated involvement of AMPKα‐BECN1 and ATG5/ATG7/NCOA4 pathways, in tandem with DEX, induces both autophagy and ferroptosis. This study sheds light on the potential adverse effects associated with DEX in the management and treatment of preterm birth‐related pregnancy disorders, thereby enhancing our current understanding of DEX‐related toxicity and fostering improved biomedical applications of DEX.

## Author Contributions

**Junlei Lu:** conceptualization (equal), data curation (equal), project administration (equal), validation (equal), writing – original draft (equal). **Xinyun Huang:** data curation (equal), project administration (equal), software (equal), validation (equal), writing – original draft (equal). **Yuan Xu:** conceptualization (equal), formal analysis (equal), methodology (equal), resources (equal), software (equal). **Qiaoping Xu:** formal analysis (equal), funding acquisition (equal), methodology (equal), resources (equal). **Hongkai Shang:** conceptualization (equal), formal analysis (supporting), funding acquisition (equal), investigation (equal), project administration (equal), supervision (equal), validation (equal), visualization (equal), writing – review and editing (equal).

## Ethics Statement

This study was approved by the Ethics Committee of Affiliated Hangzhou First People's Hospital, Zhejiang University School of Medicine (No. 2020102‐01), with written informed consent from each participant.

## Conflicts of Interest

The authors declare no conflicts of interest.

## Supporting information


Data S1.

## Data Availability

The data that support the findings of this study are available from the corresponding author upon reasonable request.
