# Supplementary material for: Dexamethasone Promotes Autophagy Dependent Ferroptosis of Placental Trophoblast Cells Through GRα
Source: J Cell Mol Med. 2025 Jul 7;29(13):e70613. doi: 10.1111/jcmm.70613 (PMC12235054; doi:10.1111/jcmm.70613)
Supplement: Supplementary file 1 — Data S1. [file JCMM-29-e70613-s001.docx]

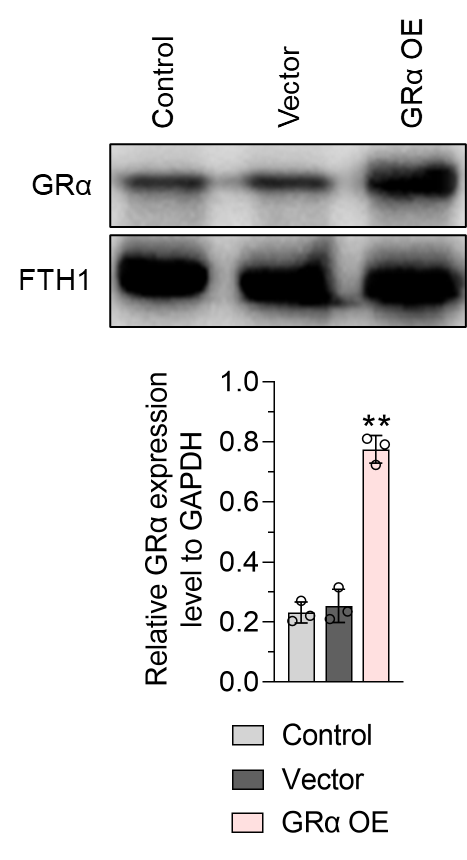


**Supplement data 1 (Figure S1).** The expression of GRα (vector effect verification) was determined by Western Blot. Data shown in the bar chart are presented as the Mean ± SD. Inter-group statistical analysis was performed by one-way ANOVA. ** represent p<0.01 VS. Vector.


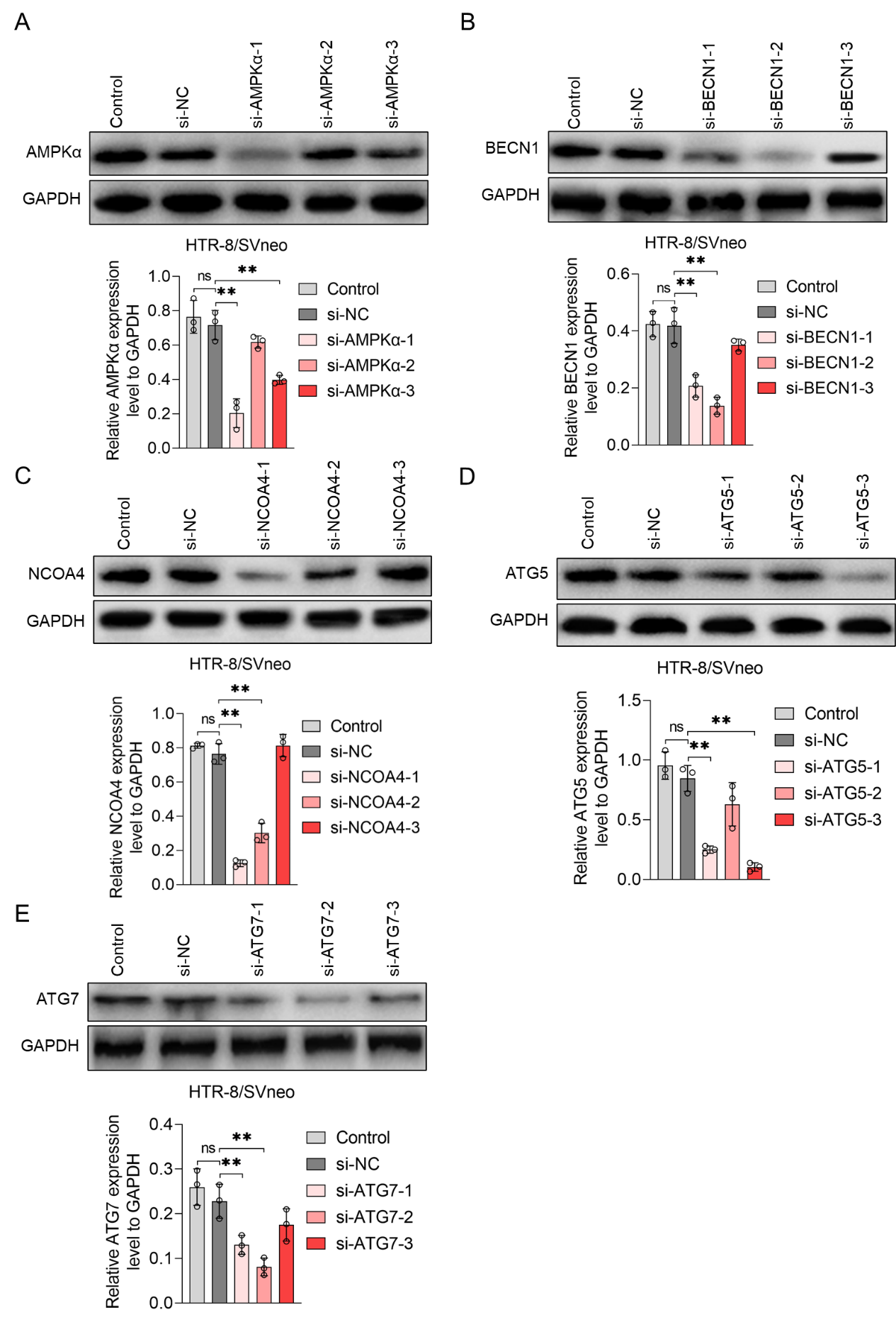


**Supplement data 2 (Figure S2).** The protein levels of AMPKα, BECN1, NCOA4, ATG5 and ATG7 were measured by Western Blot (vector effect verification). (A) The protein expression level of AMPKα (siRNA effect verification) was determined by Western Blot. (B) The proteins level of BECN1 was measured by Western Blot (siRNA effect verification). (C) ATG5 level was examined by Western Blot (siRNA effect verification). (D) Measurement of ATG7 level by Western Blot (siRNA effect verification). (E) NCOA4 level was measured by Western Blot (siRNA effect verification). Data shown in the bar chart are presented as Mean ± SD. Inter-group statistical analysis was performed by one-way ANOVA. Ns represent p>0.05, and ** represent p<0.01.
